# Mapping a sustainable approach: biosynthesis of lactobacilli-silver nanocomposites using whey-based medium for antimicrobial and bioactivity applications

**DOI:** 10.1186/s12934-024-02428-8

**Published:** 2024-07-06

**Authors:** E. B. El.Fadly, A. S. Salah, B. Abdella, A. Al Ali, H. AlShmrany, A. M. ElBaz, N. S. Abdelatty, E. F. Khamis, O. F. Maagouz, M. A. Salamah, M. N. Saleh, H. K. Sakr, M. A. El-Kemary

**Affiliations:** 1 Department of Dairy Sciences, Faculty of Agriculture, Kafrelsheikh University, Kafrelsheikh, Egypt; 2 Institute of Nanoscience and Nanotechnology, Kafrelsheikh University, Kafrelsheikh, Egypt; 3 Department of Aquaculture, Faculty of Aquatic and Fisheries Sciences, Kafrelsheikh University, Kafrelsheikh, 33516 Egypt; 4 Institute of Aquaculture, Faculty of Natural Sciences, University of Stirling, Stirling, FK9 4LA UK; 5 Faculty of Aquatic and Fisheries Sciences, Kafrelsheikh University, Kafrelsheikh, 33516 Egypt; 6 Department of Clinical Laboratory Sciences, Faculty of Applied Medical Sciences, University of Bisha, 255, Al Nakhil, 57714 Bisha, Saudi Arabia; 7 Department of Medical Laboratory Sciences, College of Applied Medical Sciences, Prince, Sattam Bin Abdulaziz University, 11942 Alkharj, Saudi Arabia; 8 Dairy Microbiology Research Department, Agriculture Research Center, Animal Production Research Institute, Giza, 12611 Egypt; 9 Department of Food Science and Nutrition, College of Biosystems Engineering and Food Science, Zhejiang University, Hangzhou, 310058 China; 10 Dairy Chemistry Research Department, Agriculture Research Center, Animal Production Research Institute, Giza, 12611 Egypt; 11 Agricultural Research Center, Food Technology Research Institute, Giza, 12611 Egypt; 12 Nile Valley University, Fayum, Egypt

**Keywords:** Synergism, sustainable whey- based medium, Silver nanocomposites biosynthesis, lactobacilli, Spore-formers, multidrug resistant bacteria, antimicrobial Activity, antioxidant activity

## Abstract

This study explores a sustainable approach for synthesizing silver nanocomposites (AgNCs) with enhanced antimicrobial and bioactivity using safe *Lactobacillus* strains and a whey-based medium (WBM). WBM effectively supported the growth of *Lactobacillus delbrueckii* and *Lactobacillus acidophilus*, triggering a stress response that led to AgNCs formation. The synthesized AgNCs were characterized using advanced spectroscopic and imaging techniques such as UV‒visible, Fourier transform infrared (FT-IR) spectroscopy, transmission electron (TEM), and scanning electron microscopy with energy dispersive X-ray analysis (SEM–Edx). *Lb acidophilus*-synthesized AgNCs in WBM (had DLS size average 817.2–974.3 ± PDI = 0.441 nm with an average of metal core size 13.32 ± 3.55 nm) exhibited significant antimicrobial activity against a broad spectrum of pathogens, including bacteria such as *Escherichia coli* (16.47 ± 2.19 nm), *Bacillus cereus* (15.31 ± 0.43 nm), *Clostridium perfringens* (25.95 ± 0.03 mm), *Enterococcus faecalis* (32.34 ± 0.07 mm), *Listeria monocytogenes* (23.33 ± 0.05 mm), methicillin-resistant *Staphylococcus aureus* (MRSA) (13.20 ± 1.76 mm), and filamentous fungi such as *Aspergillus brasiliensis* (33.46 ± 0.01 mm). In addition, *Lb acidophilus*-synthesized AgNCs in WBM exhibit remarkable free radical scavenging abilities, suggesting their potential as bioavailable antioxidants. These findings highlight the dual functionality of these biogenic AgNCs, making them promising candidates for applications in both medicine and nutrition.

**Graphical Abstract:**

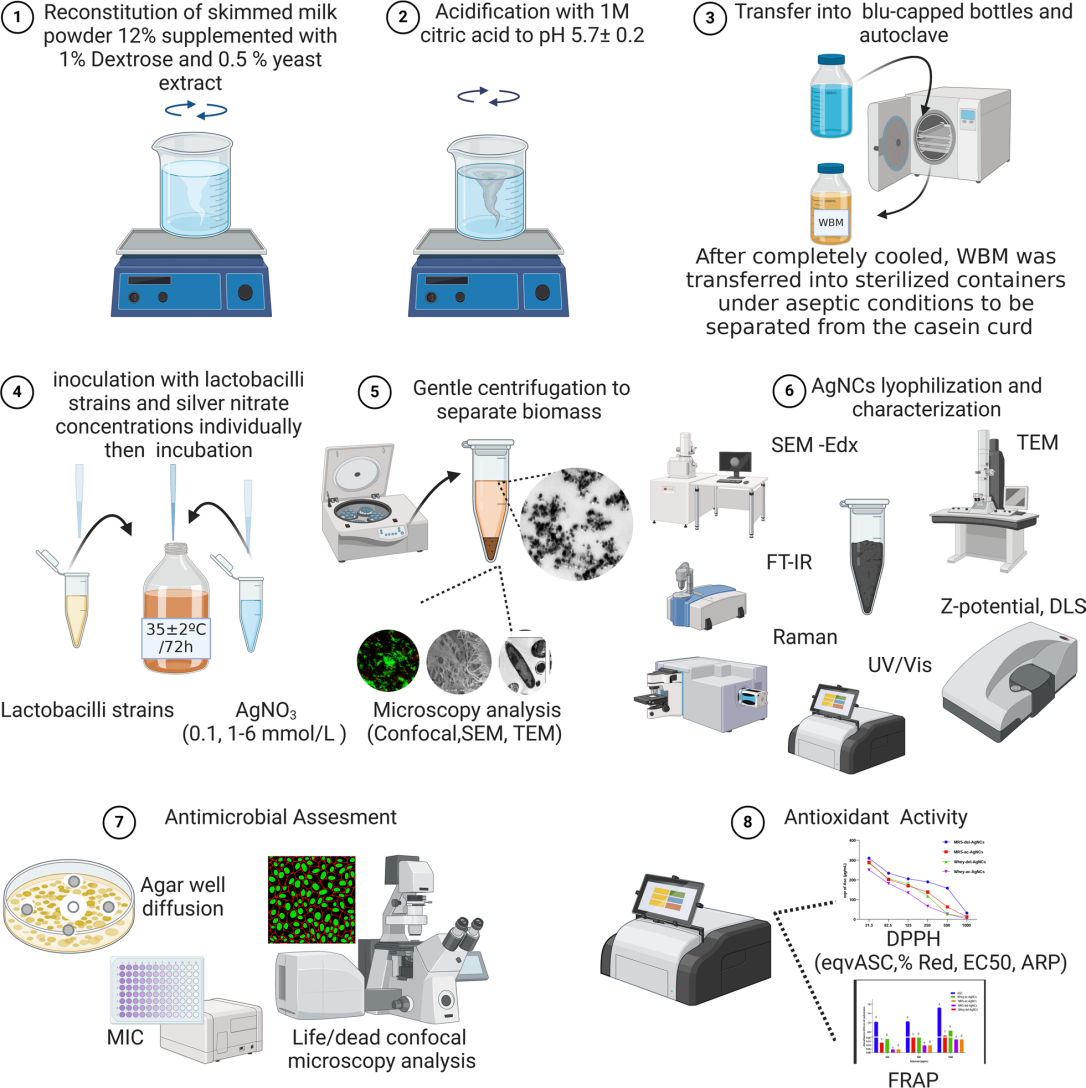

## Introduction

Biological nanoparticle synthesis methods offer valuable and eco-friendly alternatives to traditional chemical and physical methods. Among these biological methods, the use of lactobacilli, a genus of lactic acid bacteria (LAB) that are generally regarded as safe (GRAS) by the FDA, has gained prominence [1–3]. Lactobacilli are not only beneficial for boosting immune and digestive health, but are also capable of producing metal nanoparticles like gold, silver, and zinc offering a commercial and environmentally friendly strategy to combat pathogens [4–6]. These nanoparticles, particularly silver nanoparticles (AgNPs), synthesized using lactobacilli, have shown potent antimicrobial efficacy against diverse microorganisms, including yeasts, bacteria, fungi, and parasites, which is crucial in the era of rising antimicrobial resistance [7–9].

The effectiveness of AgNPs is significantly influenced by their size, concentration, and the biological approaches used for their synthesis [10–12]. This becomes particularly crucial in light of the emergence of multidrug-resistant (MDR) pathogenic bacterial strains [7, 13, 14–]. In addition, the synthesis environment plays a critical role in the properties of nanoparticles. Whey, a byproduct of the dairy industry historically considered a pollutant, has found significant applications in nanoparticle biosynthesis. As a nutritive medium, whey enhances the growth and metabolic activity of lactobacilli, facilitating the production of highly stable and well-characterized nanoparticles. Research on whey as a growth medium highlights the adoption of ecologically friendly substrates that reduce environmental damage typically caused by chemical synthesis methods [16–19].

This study explores the effects of the metabolic activities of Lactobacillus strains in a whey-based medium on the biosynthesis efficiency of silver nanocomposites (AgNCs). In addition we studied the synergistic effect of theis metabites as capping agent to scale up the antimicrobial activity of AgNCs, which are recognized for their stability and effectiveness against multidrug-resistant (MDR) pathogens and endospore formers. Furthermore, the study compares the comprehensive physicochemical properties of AgNCs with conventional antibiotic treatments and examines the antioxidant activity of AgNCs with organic coatings derived from different Lactobacillus strains.

## Materials and methods

### Microbial strains

Lactobacilli cultures used in this study were *Lactobacillus delbrueckii* subsp *bulgaricus* ATCC 7995 (*Lb delbrueckii*), *Lactobacillus acidophilus* ATCC 4356 (*Lb acidophilus*), *Lactobacillus casei* subsp. *casei* ATCC 393 (*Lb casei*), *Lactobacillus plantarum* subsp. *plantarum* ATCC 14917 (*Lb plantarum*), and *Lactobacillus rhamnosus* ATCC 7469 (*Lb rhamnosus*)*.* The indicator pathogenic strains were *Escherichia coli* ATCC 8739 (*E coli*), *Staphylococcus aureus* MRSA ATCC 43300 (*Staph aureus* MRSA), *Bacillus cereus* ATCC 10876 (*B cereus*), *Clostridium perferinges* ATCC 13124 (*C perferinges*), *Enterococcus faecalis* ATCC 51299 (*E faecalis*), *Listeria monocytogenes* ATCC 11994 (*L monocytogenes*) and *Aspergillus brsillensis* ATCC 16404 (*Asp brsillensis*).

### Materials

Silver nitrate (AgNO_3_) (Merck 654833, Germany), glacial acetic acid (BDH UN2789, France), methanol ((Fisher UN1230, UK), ethanol (SERVA UN1170, Germany), acridine orange (Alfa Aether, L13159, Germany), ethidium promise (Alfa ether, J66192, Germany), resazurin (BDH, 59506400, England), 2,2-diphenyl-1-picrylhydrazyl (DPPH) (Sigma Aldrich, D9132, Germany), potassium ferric cyanide (Alfa Aesar, 33557, Germany), trichloroacetic acid (LOBA Chemical, L327632002, India), and ferric chloride (Chem-Lab,0.0909.1000, Belgium).

### Media for synthesizing the silver bio nanocomposite

Two different media were used for the growth of lactobacilli strains and the production of AgNCs, namely, MRS (DeMan, Rogosa, and Sharpe) broth medium (Oxoid Code CM0359) [20] and a whey-based medium (WBM) prepared using a simple acidification method, where 12 g of skim milk powder was reconstituted in 100 mL of distilled water and fortified with 0.5% yeast extract (Sigma Aldrich, Germany) and 1% glucose (Sigma Aldrich, Germany). After full solubilization, the solution was acidified using citric acid (1 M) to a pH of 5.7 ± 0.2 [21]. The acidified skim milk was autoclaved at 121 °C for 15 min to sterilize and separate the whey. After completely cooling, the WBM was carefully transferred into sterilized containers under aseptic conditions for separation from the casein curd. For antimicrobial activity, two media were utilized, Mueller Hinton broth (MHB) (Oxoid code CM0405) at pH 7.3 ± 0.1 for antibacterial activity [22] and malt extract agar (MEA) (Oxoid code CM0057) at pH 5.4 ± 0.2 at 25 °C for the antifungal activity test [23]

### Determination of AgNO_3_ tolerance of lactobacilli strains

To determine the tolerability of each *Lactobacillus* strain to AgNO_3_, individual lactobacilli strains were cultured overnight in MRSB and WBM with 0.1 mmol AgNO_3_ to elicit the ability of the lactobacilli strains to adapt to higher concentrations of AgNO_3_. After overnight culture, 0.5 mL (OD600 ~ 0.08–0.1) of fresh medium supplemented with different concentrations of AgNO_3,_ namely, 1, 2, 3, 4, 5, or 6 mmol AgNO_3,_ was inoculated into 10 mL of fresh medium. The inoculated tubes were incubated at 37 °C under aerobic conditions for 72 h. The OD600 of each tube was measured every 2 h for the first 12 h and then every 12, 24, 36, 48, and 72 h using a double beam Cary 3500 UV‒Vis spectrophotometer (Agilent, USA) in absorbance mode. The experiments were performed in triplicate [24]. The concentration and strain selection were predicated on a 24 h incubation period (first point). The tolerance of the lactobacilli strains to AgNCs accumulation after 72 h (the end of the incubation period) was subsequently investigated by employing a double-beam Cary 3500 UV‒Vis spectrophotometer (Agilent, USA), absorbance mode, comprehensive microscopy analyses, confocal, TEM, and SEM imaging techniques.

### Confocal microscopy analysis

To test cellular viability after 72 h of incubation with AgNO_3_, confocal microscopy was performed via acridine orange/ethidium bromide (AO/EB) at a final concentration of 0.12/0.4 µg/mL [25]. The lactobacilli biomass was fixed using paraformaldehyde (4%), stained with AO/EB, incubated at room temperature for 5 min in the dark, and centrifuged, and the remaining supernatant was discarded to eliminate the unbound dye. Next, the cell pellet was resuspended in a small volume of 1X-PBS. Control samples were prepared from untreated media supplemented with AgNO_3_. All the experiments were performed in a dark room to avoid photo-bleaching of the dyes. Live/dead cell count visualization was carried out by using a Leica DMi8 confocal microscope (Leica, Wetzlar, Germany) with a 63 × oil immersion objective, and images were collected. The captured images were analyzed using ImageJ software [26].

### Biosynthesis of silver nanocomposites (AgNCs)

AgNCs were biosynthesized using a tolerable concentration of AgNO_3_. Then, the preadapted lactobacilli strains were individually inoculated with 0.5 ml of OD600 0.08–0.1. After 72 h of incubation at 37 °C, the individual strains of biomass in both media were filtered through a sterilized 0.22 µm membrane filter using a vacuum pump (IKAMVP basic, Imlab, France) filtration unit. The collected biomass was resuspended in 1 ml of 1X- PBS and then fixed immediately in paraformaldehyde solution (4%) for microscopy analysis. Then, the cell-free media filtrate containing AgNCs was centrifuged at 10,784 × g at room temperature for 30 min for collection and concentration. Then, the nanocomposites were purified 5 times by centrifugation at 10,784 × g at room temperature for 15 min with sterilized deionized distilled water. Additionally, to remove unreacted silver ions and low-molecular-weight metabolites, three days of 3.5 kDa cut-off dialysis was carried out [27], followed by freeze-drying, characterization, and subsequent bioavailability studies, including antimicrobial and antiradical methods [28].

### Characterization of the AgNCs

The absorption spectra of the biosynthesized bioactive AgNCs in WBM and MRSB media were obtained using a double beam Cary 3500 UV‒Vis spectrophotometer (Agilent) operating in a scanning mode in the range of 200–800 nm. Zeta analysis and dispersion stability and size distribution profile (DLS) measurements were carried out using a Brookhaven zeta potential/particle size analyzer [11]. The surface morphologies of the bacterial strains were studied via a field emission scanning electron microscope (ZEISS Sigma 500 VP FE-SEM) instrument equipped with a dispersive EDX unit. To study the size, shape, and morphology of the AgNPs, high-resolution transmission electron microscopy (HR-TEM) was carried out using (JEM-2100, JEOL) [29]. Fourier transform infrared spectroscopy (FTIR) (JASCO, Tokyo, Japan, model no AUP1200343) was used to determine the molecular structure of the nanocomposite surface at 500–4000 cm^−1^ using the KBr pellet method [12]. Finally, micro-Raman spectroscopy was performed using an alpha300 R confocal microscope (WITec GmbH). An A100X objective microscope was used to focus the laser beam on the sample to a size of ~ 300 nm (with a diffraction limit of 532 nm). A 532 nm green laser excitation was used. The spectrometer wavenumber scale was calibrated using a 521rel instrument. cm.^−1^ line for Si. [30]

### Antimicrobial activity of the AgNCs

#### Agar well diffusion assay

The agar well diffusion method was used to measure antibacterial and antifungal activity [31, 32]. Antibacterial activity was carried out as follows: 30 mL of MHA in a 150  mm Petri dish was surface inoculated with 100 µL (evenly spread using sterilized Q-tip cotton swabs) of the indicator strain (OD600 0.08–0.1) for each strain. Then, nine wells were made in each plate using sterilized tips (diameter 0.5 mm). The lyophilized silver nanocomposites (AgNCs) were re-suspended in sterilized deionized water at 100 µg/mL, sonicated in an ice bath for 15 min to prevent overheating and potential degradation of the nanocomposites, vigorously vortexed to ensure further disaggregation, and homogeneous dispersion of the nanoparticles in solution. This was performed immediately before aliquots of 100 µl were drawn using an automatic micropipette for inclusion in the agar wells. To maintain a consistent dispersion of the nanoparticles throughout the procedure, the suspension was vortexed repeatedly prior to each pipetting action. Finally, 100 µL of gentamicin (8 µg/mL) was added in the middle well as a positive control. Six negative controls were used: uninoculated whey medium, *Lactobacillus* individual strain-inoculated whey medium, WBM with silver nitrate (4 mmol), uninoculated MRS, *Lactobacillus* individual strain-inoculated MRS, and uninoculated MRSB with silver nitrate (4 mmol). All the plates were incubated under their optimum conditions. The antifungal activity was determined by inoculating 30 mL of MEA with 100 µL of known fungal spores (1 × 10^4^ spores/mL) of *Asp brasiliensis*. Then, the inoculated medium was poured into a 150 mm Petri dish and settled at room temperature until solidification. Nine holes with a diameter of 5 mm were punched using sterile tips, and 100 µl of the AgNCs biosynthesized on MRSB or WBM (100 µg/mL) was subsequently added to each of the two wells. The other six wells were filled with the aforementioned negative controls, and the center well was filled with 100 µL of terbinafine (0.4 µg/mL) as the positive control in the middle well. The plates were incubated aerobically at 18 °C for 4 days. All of the antibacterial and antifungal experiments were conducted in  replicates. By the end of the incubation period, the inhibition zones around the wells were measured and presented in millimeters.

#### Minimum inhibitory concentrations (MICs)

MICs were determined following the CLSI M07-A10 and CLSI M100 guidelines. Briefly, in 96-well plates, twofold dilutions of 100 µg/L AgNCs were added to 100 µL of MHB, followed by 100 µL of MHB from *E coli* (Gve-), *Staph aureus* MRSA (Gve +), *B cereus* Gve + (aerobic)*,* and *C perferinges* Gve + (anaerobic) endospore former microorganisms, with an absorbance ranging from 0.08–0.1 nm at 600 nm for each strain in individual experiments carried out in 96-well microplates after overnight optimum incubation. The absorbance was measured at 620 nm using a BMG LABTECH^®^-FLUOstar Omega microplate reader (Ortenberg, Germany). Gentamicin (8 µg/L) was used as a positive control [33]. 20 µL of Resazurin stain solution (1 mg/L) was added to each well to confirm the MIC visually [34]. All experiments were conducted in triplicate.

#### Confocal microscopy analysis

The confocal microscopy assay was chosen to investigate the ability of the synthesized AgNCs to serve as efficient antimicrobial agents. This method investigated the viability of *Staph aureus* MRSA and *E coli* cells exposed to AgNCs at concentrations above the MIC. The cells (1 × 10^6^ CFU) were treated with AgNCs and incubated for 24 h at 37 ± 2 °C. Next, the cells were stained with two fluorochromes, acridine orange (0.12 µg/mL) and ethidium bromide (0.4 µg/mL). Untreated *Staph aureus* MRSA and *E coli* cells served as the control to determine the background levels of viability under the experimental conditions. The live and dead cells were visualized using a confocal microscope (Leica DMi8; Leica, Wetzlar, Germany) equipped with a 63 × oil immersion objective, and images were collected [32]. The images were captured and analyzed using ImageJ 1.49v software [26].

### Antioxidant activity of the AgNCs

Two separate experiments were carried out to delve deep into the antioxidant activity of AgNCs. First, serial twofold dilutions (31.3, 62.5, 125, 250, 500, and 1000 µg/mL) were used to study the bioavailability of AgNCs, which are expressed as equivalent µg/mL acid as a well-known antioxidant substrate. The results of the free radical scavenging assay (RSA) and determination of the antiradical parameters of the AgNCs biosynthesized from WBM and MRSB are expressed as DPPH-reduction (%) data. The EC50 values (the concentration of the AgNCs that could reduce DPPH to 50% of that of the WBM or MRSB nanocomposites) were estimated via nonlinear regression. Moreover, the other antioxidant parameters of the studied nanocomposites were theoretically calculated based on the following equation: ARP (antiradical power) = 1/EC50 [28, 32]. Second, as confirming evidence, a ferric-reducing power assay (FRAP) was carried out with three concentrations (250, 500, and 1000 µg/mL) of FA compared to those of ascorbic acid [35]. All experiments were conducted in triplicate.

### Statistical analysis

Prior to statistical analysis and modeling, the data were tested for normality and variance equality assumptions using Shapiro‒Wilk’s and Levene’s homogeneity tests, respectively. A P value less than 0.05 indicated statistical significance (*p* < 0.05). After 24 h of incubation on each medium in the presence of different concentrations of AgNO_3,_ the strain growth, in terms of log CFU/mL, was evaluated for each medium using one-way ANOVA to determine the best AgNO_3_ concentration. For confirmation, interpolation of the optimum tolerable concentration of AgNO_3_ for the selected strains proliferating on both media and the best-fit models was conducted using GraphPad Prism version 9.4.0 (GraphPad Software, Inc., San Diego, USA) to run different polynomial models. Then, the best-fit model was selected through the results of the R-squared test, the sum of squares F test, the root means square error (RMSE), and the Akaike information criterion (AIC). The best-fit model equations for strain growth are shown in each figure. For antimicrobial and antifungal evaluation of the bio silver nanocomposites, normalized data of the inhibition zones against some selected pathogens were statistically analyzed using one-way ANOVA for each profiled pathogen, followed by post hoc multiple comparison tests (Tukey’s HSD test). Moreover, similarities in the antimicrobial efficacies of the nanocomposites against pathogens were visualized using standardized PCA with Rstudio (version 4.0.3). The free radical scavenging activity of AgNCs (at 3 concentrations) in MRSB and WBM compared to that of ascorbic acid was analyzed using one-way ANOVA for each investigated substrate, followed by Tukey’s HSD test.

## Results

### Determination of AgNO_3_ tolerance in lactobacilli cultures

The viable cell count (log CFU) for the lactobacilli cultures was assessed after 24 h of incubation with and without AgNO_3_. Notably, the highest growth of the lactobacilli strains was observed at a concentration of 4 mmol following a prestimulation period overnight at 0.1 mmol AgNO_3_. Among the five strains tested, *Lb delbrueckii* and *Lb acidophilus* had the most favorable growth characteristics, in terms of CFU/mL, when cultured in both whey-based medium (WBM) and MRSB (Table 1).

**Table 1:** Log CFU/mL of *Lactobacillus* strains after incubation (24 h) with silver nitrate concentrations

	*Lb delberuckii*		*Lb acidophilus*		*Lb casei*		*Lb plantarum*		*Lb rhamnosus*	
	Whey	MRSB	Whey	MRSB	Whey	MRSB	Whey	MRSB	Whey	MRSB
0*	**3.96 ± 0.01**^a^	**3.47 ± 0.02**^a^	**4.57 ± 0.02**^a^	**3.80 ± 0.05**^a^	**2.89 ± 0.47**^a^	**1.80 ± 0.05**^a^	**3.30 ± 0.17**^a^	**2.48 ± 0.19**^a^	**3.01 ± 0.07**^a^	**2.36 ± 0.15**^a^
0.1	1.01 ± 0.05^f^	0.92 ± 0.02^f^	1.14 ± 0.05^h^	0.95 ± 0.04^g^	0.67 ± 0.15^f^	0.46 ± 0.12^e^	0.98 ± 0.07^f^	0.75 ± 0.04^f^	1.08 ± 0.48^e^	0.56 ± 0.06^f^
1	1.27 ± 0.05^ef^	1.17 ± 0.01^e^	1.54 ± 0.04^g^	1.17 ± 0.01^f^	1.04 ± 0.03^e^	0.95 ± 0.04^c^	1.19 ± 0.07^e^	1.05 ± 0.05^e^	1.01 ± 0.02^e^	0.86 ± 0.07^e^
2	1.40 ± 0.08^e^	1.32 ± 0.03^d^	1.87 ± 0.11^f^	1.47 ± 0.03^e^	1.17 ± 0.01^d^	1.01 ± 0.09^bc^	1.58 ± 0.14^d^	1.26 ± 0.07^d^	1.29 ± 0.20^d^	1.03 ± 0.02^d^
3	2.90 ± 0.02^b^	2.16 ± 0.04^b^	3.22 ± 0.05^c^	2.16 ± 0.04^c^	2.03 ± 0.03^b^	1.15 ± 0.04^b^	2.08 ± 0.05^c^	1.79 ± 0.15^c^	1.62 ± 0.24^c^	1.35 ± 0.06^c^
**4**	**3.80 ± 0.04**^ab^	**3.47 ± 0.05**^a^	**4.11 ± 0.06**^b^	**3.05 ± 0.00**^b^	**2.14 ± 0.04**^b^	**1.25 ± 0.00**^b^	**2.80 ± 0.02**^c^	**2.51 ± 0.05**^b^	**2.15 ± 0.04**^b^	**1.85 ± 0.05**^b^
5	2.44 ± 0.12^c^	2.26 ± 0.04^b^	2.88 ± 0.05^d^	2.20 ± 0.07	1.88 ± 0.06^bc^	1.01 ± 0.02^bc^	2.33 ± 0.04^b^	2.00 ± 0.00^c^	1.61 ± 0.05^c^	1.30 ± 0.05^c^
6	1.60 ± 0.04^d^	1.37 ± 0.02^c^	2.59 ± 0.05^e^	1.81 ± 0.10^d^	1.59 ± 0.05^c^	0.81 ± 0.11^d^	1.37 ± 0.22^de^	1.05 ± 0.02^e^	1.01 ± 0.02^e^	0.80 ± 0.03^e^

^*^Lactobacilli cultures incubated for 24 h with different AgNO_3_ concentration (0,0.1, 1–6 mmol) individualy in WBM and MRSB media. (mean ± SD)

**Fig. 1:**
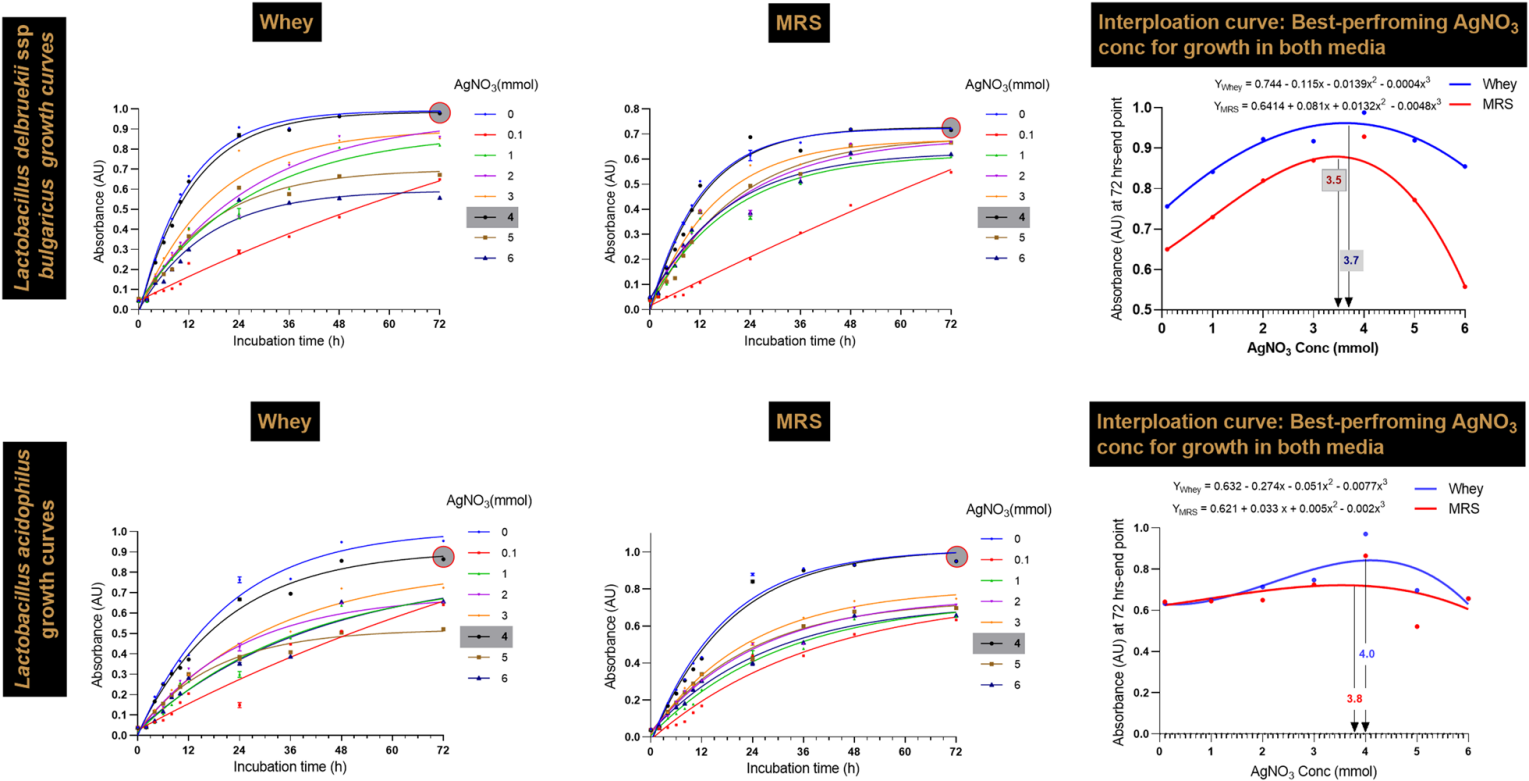
Growth of *Lb. delbrueckii* and *Lb acidophilus*, best fit ANCOVA, and interpolation curves after incubation with AgNO_3_

The growth rates at 2 h intervals for 12 h, then 24 36 h, 48 h, and 72 h were determined for the top two strains, *Lb delbrueckii* and *Lb acidophilus.* The best-performing AgNO_3_ concentrations for the best-tested lactobacilli cultures were subsequently adjusted after 72 h of incubation. *Lb delbrueckii* and *Lb acidophilus* exhibited optimal growth at 3.5 and 3.8 mmol, respectively, in MRSB, while in whey-based medium (WBM), the optimal concentrations were 3.7 and 4.0 mmol for both strains, respectively (Fig. 1).

**Fig. 2:**
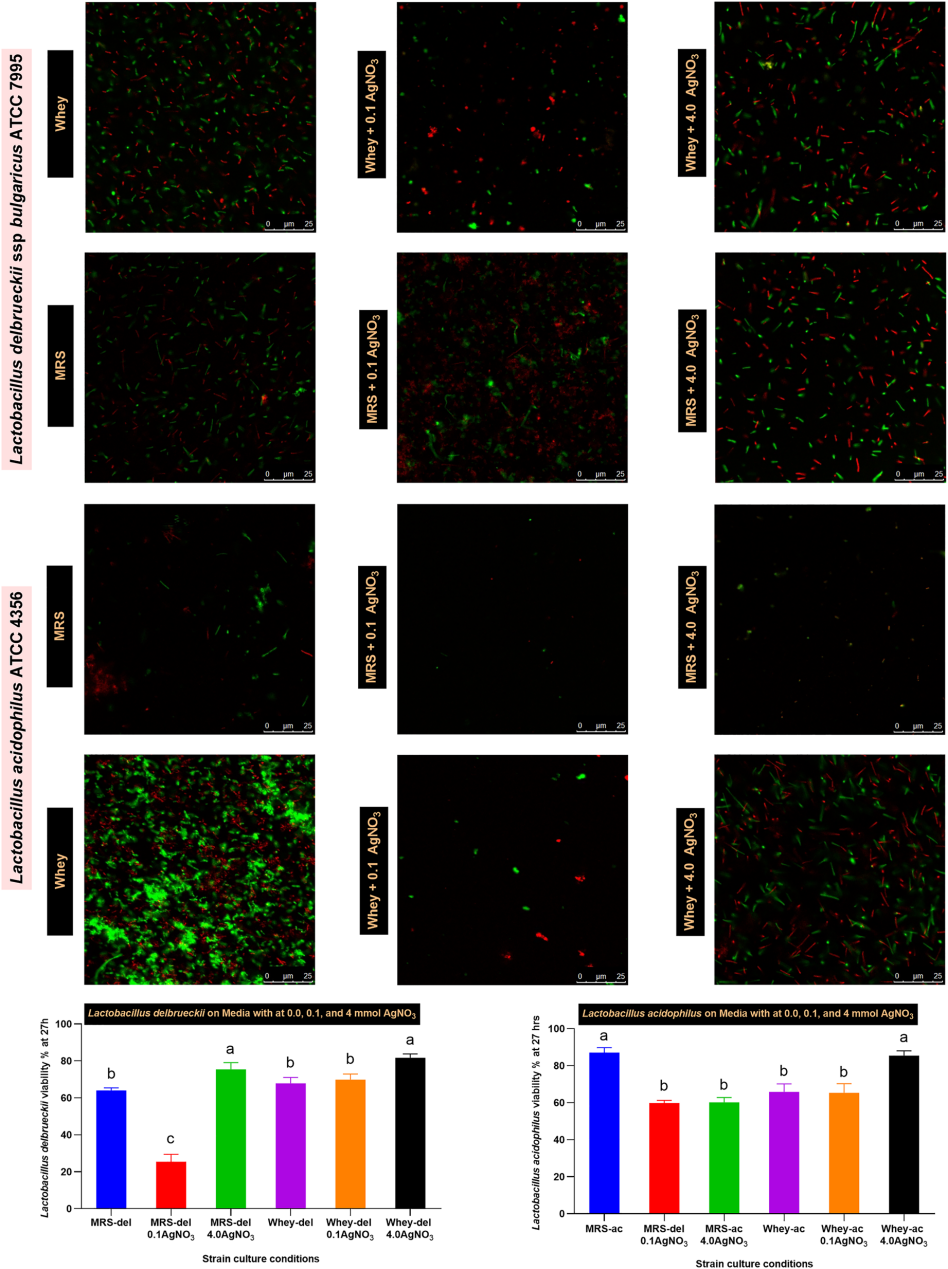
Sublethal silver nitrate impact on *Lb delbrueckii* and *Lb acidophilus* determined using acridine orange/ethidium bromide

To evaluate the impact of prestimulation with a sublethal AgNO_3_ stressor on the viability and resilience of *Lb delbrueckii* and *Lb acidophilus*. Prestimulation with 0.1 mmol AgNO_3_, followed by exposure to 4 mmol AgNO_3_, enhanced cell resilience, as evidenced by an increase in green fluorescence (indicating viability) compared to red fluorescence. Cells that were not prestimulated were more adversely affected. The results confirmed that whey-based medium (WBM) enhanced survival more than MRSB, underscoring its potential significance in this study (Fig. 2).

**Fig. 3:**
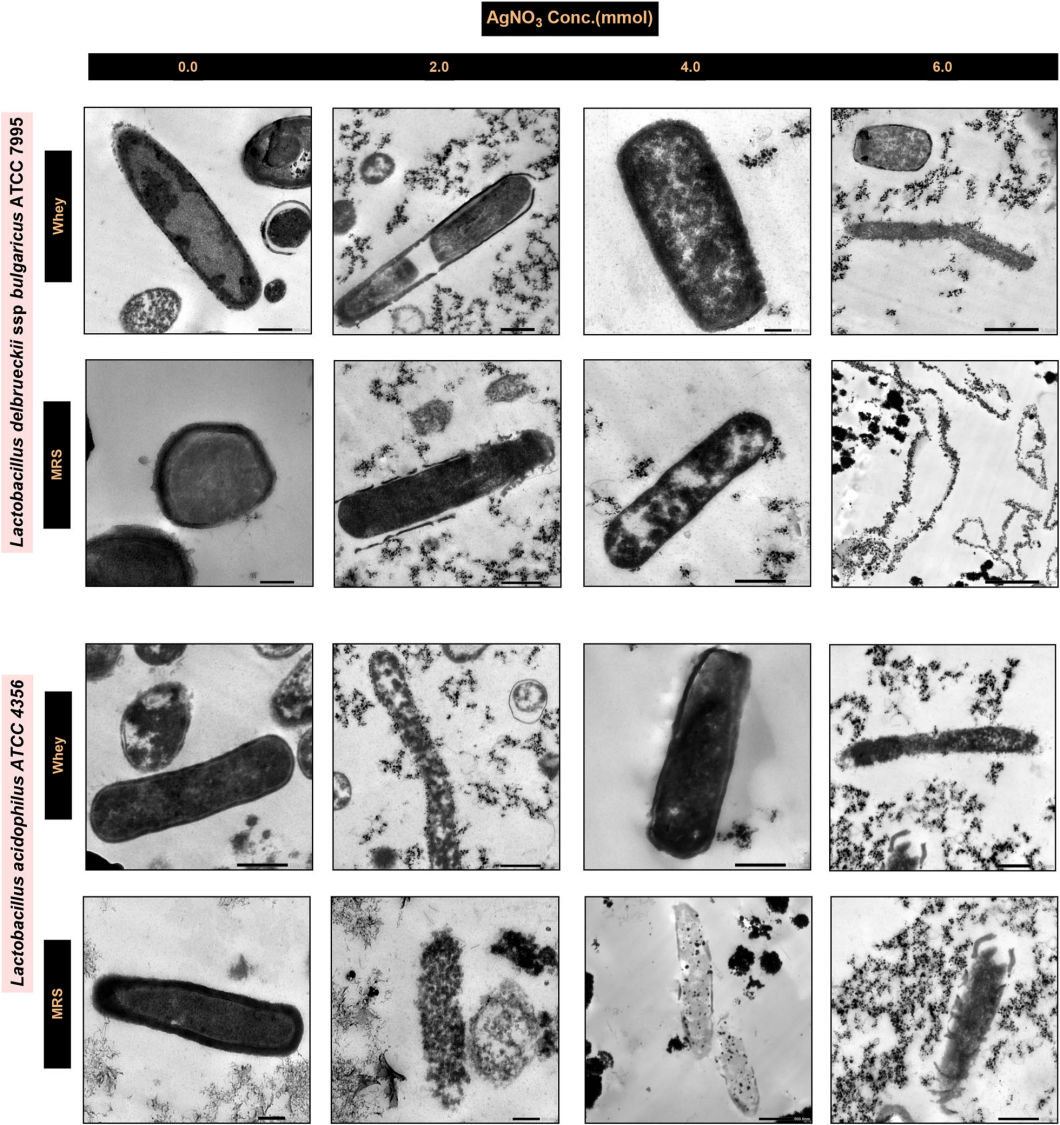
HR-TEM images of *Lb delbrueckii* and *Lb acidophilus* incubated in MRSB and WBM with AgNO_3_ concentrations

Finally, high-resolution transmission electron microscopy (HR-TEM) images of *Lb delbrueckii* and *Lb acidophilus* cultured in both media for 72 h confirmed the previously mentioned results, where the presence of AgNO_3_ concentrations greater than or less than 4 mmol significantly reduced the growth of the lactobacilli cultures. Thus, an AgNO_3_ concentration of 4 mmol was identified as the key concentration at which most strains were tolerable, facilitating comparisons among strains. Moreover, the TEM images demonstrated that in the MRSB, all the strains exhibited an aggressive response at the 72 h incubation endpoint, when the silver nanoparticles affected the cell walls, aggregating and penetrating the inner structure of the cells, leading to the rupture of *Lb delbrueckii* cells and deterioration of *Lb acidophilus* cells (Fig. 3).

### Characteristics of lactobacilli biosynthesized silver nanocomposites AgNCs

**Fig. 4:**
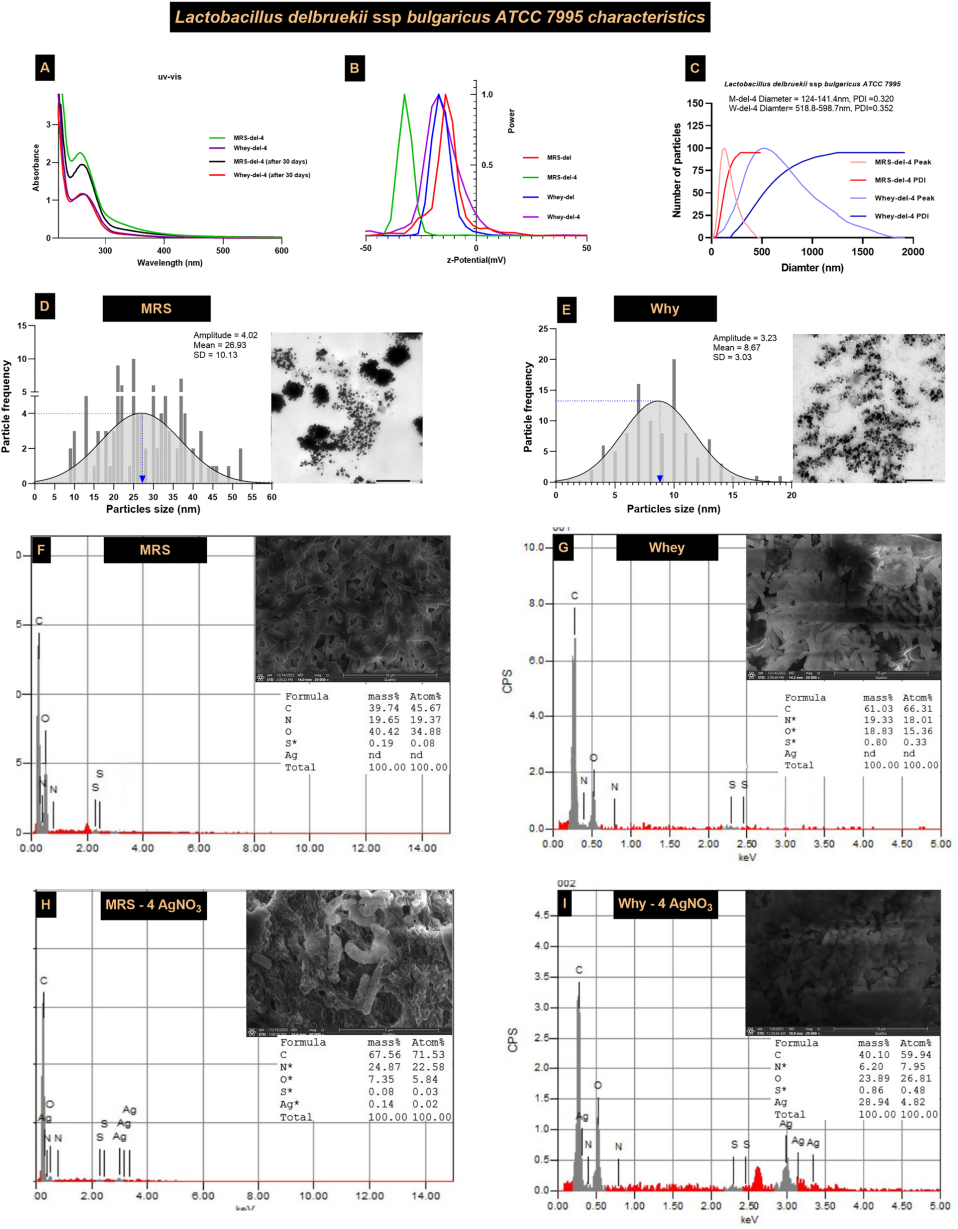
Characterization of *Lb delbrueckii* AgNCs in WBM compared to MRSB **A** UV/Vis spectra of *Lb delbrueckii* AgNCs on MRSB and WBM, **B** z-potential, **C** DLS (**D**, **E**), silver particles size distribution, and **F**–**I** SEM–EDX

Characterization of AgNCs was conducted to investigate the unique properties of the bioactive AgNCs produced by the tested lactobacilli strains. An array of analytical techniques, including spectrofluorometry, spectrophotometry, and spectroscopy, was employed. The UV/Vis spectroscopy absorption spectra of the *Lb delbrueckii* bioactive AgNCs in both MRSB and WBM ranged from 257 to 264 nm, with zeta potentials of -24.45 ± 1.54 in MRS broth and -16.84 ± 0.77 in WBM. After one month, the uv/vis peaks of AgNCs in both MRSB and WBM seemed consistent with the initial peaks, suggesting that the AgNCs were stable over time. The absence of significant peak shifts or broadening suggests minimal aggregation and negligible changes in particle size distribution, supporting the stability of nanoparticles (Fig. 4A & B). Additionally, dynamic light scattering (DLS) results indicated that the dominant diameter of the bioactive AgNCs in MRSB was between 124.0 and 141.4 nm, while in WBM, it ranged from 518.8 to 598.7 nm. High-resolution transmission electron microscopy (HR-TEM) further revealed that the morphology and metal size of the bioactive AgNCs in the MRSB were 26.93 ± 10.13 nm, while those in the WBM were smaller, at 8.67 ± 3.03 nm. The silver core was smooth, spherical, and well dispersed in WBM, whereas it was aggregated in MRS broth (Fig. 4C–E). Finally, *Lb delberuekii* exhibited better propagation in WBM than in MRSB, and the elemental composition of the bioactive AgNCs, as revealed by SEM–EDX results, was Ag = 28.94% and S = 0.86% in WBM compared to Ag = 0.14% and S = 0.08% in MRSB (Fig. 4F–I).

**Fig. 5:**
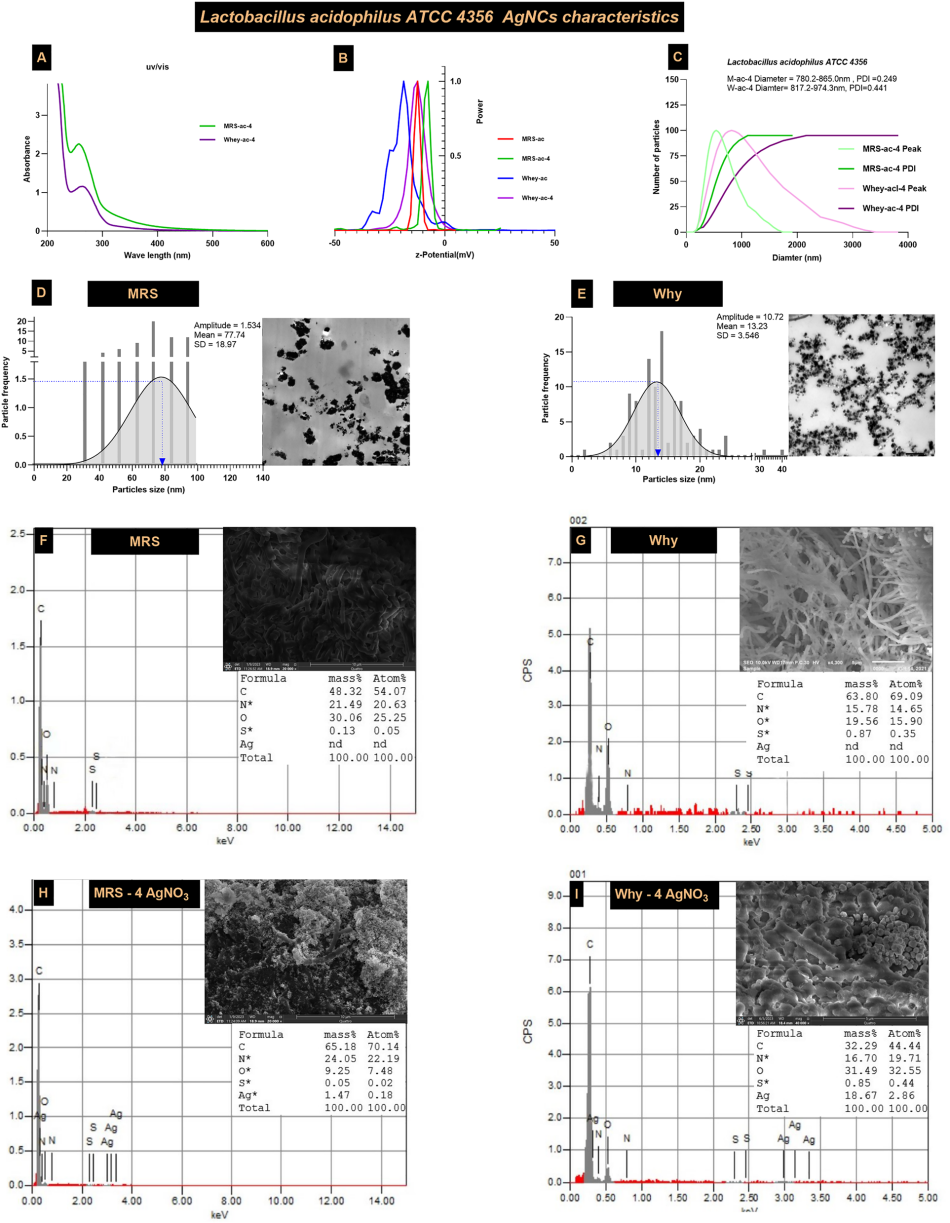
Characterization of *Lb acidophilus* AgNCs in WBM compared to MRSB **A** UV‒Vis spectra of *Lactobacillus acidophilus* AgNCs on both MRSB and WBM, **B** z-potential, **C** DLS, **D**, **E** silver nanoparticle size distribution, and **F**–**I** SEM–EDX

For AgNCs synthesized by *Lb acidophilus*, the UV/Vis absorption spectrum ranged from 258 to 264 nm for both media. The zeta potential of MRSB was − 8.41 ± 0.71, while that of WBM was − 12.61 ± 1.22. UV–vis analysis after one month revealed no shift in the wavelength of AgNC peaks for either nanocomposite, suggesting good stability of the samples (Fig. 5A & B). DLS measurements indicated that the particle size ranged from 780.2 to 885.0 nm in the MRSB and from 817.2 to 974.3 nm in the WBM. HR-TEM analysis of the particle size distribution revealed metal sizes of 77.74 ± 18.97 nm in MRSB and 13.32 ± 3.55 nm in WBM (Fig. 5C–E). The silver core was smooth, spherical, and well dispersed in WBM, in contrast to the aggregated state observed in MRSB. Additionally, *Lb acidophilus* exhibited better propagation in the WBM than in the MRSB. The elemental composition of the bioactive AgNCs, as determined by SEM–EDX, was Ag = 18.67% and S = 0.85% in WBM, compared to Ag = 0.05% and S = 1.47% in MRSB (Fig. 5F–I).

**Fig. 6:**
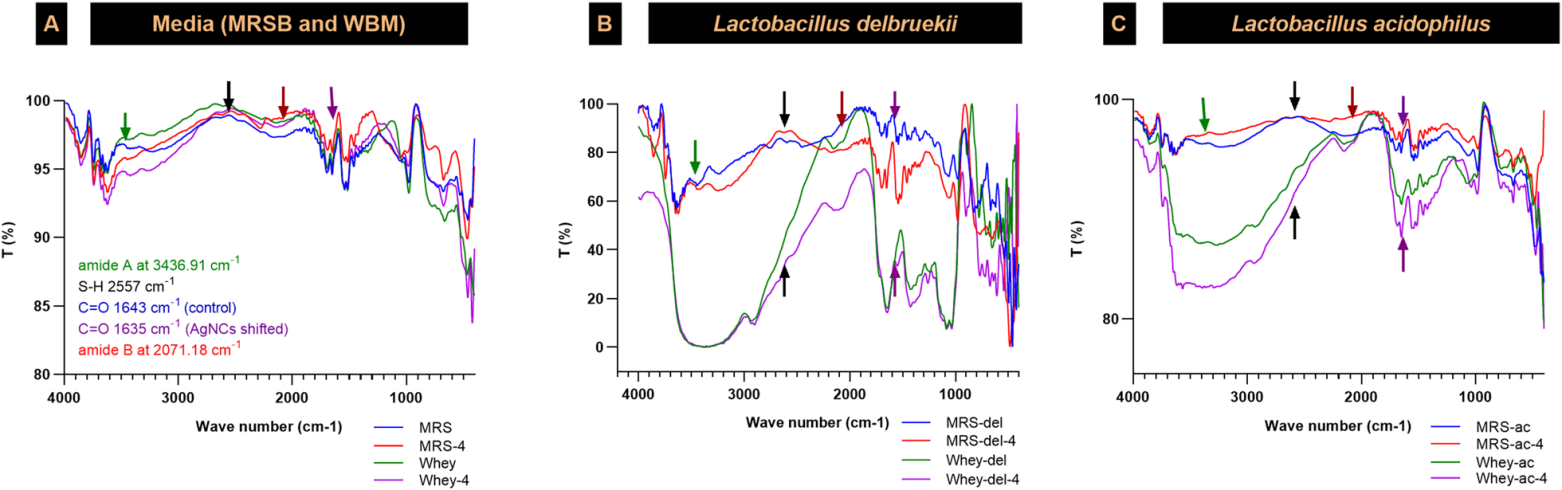
FT-IR spectra of **A** media (MRSB and WBM), **B** *Lactobacillus delbrueckii*, and **C** *Lactobacillus acidophilus*

Fourier transform infrared (FT-IR) spectroscopy maps of the two selected lactobacilli strains were obtained at the end of the 72 h incubation period on WBM and compared to those on MRSB. Among the AgNCs, the protein amide A, B, I, II, and III groups were identified. Notably, the peaks at 3436.91 cm^−1^ (amide A) and 2071.18 cm^−1^ (amide B) indicated NH vibrations. The peak at approximately 1637.62 cm^−1^ corresponded to the amide I group, indicating the broadening of the C = O stretch of the peptide bond. The amide II group, characterized by a peak at approximately 1538 cm^−1^, presented a distinct appearance. In the FT-IR spectrum, the peak corresponding to C = O carbonyl groups, typically at 1643 cm^−1^, shifted to 1635 cm^−1^ due to binding with silver, indicating interactions between the lactobacilli-produced biomolecules and the synthesized AgNCs. Additionally, the presence of thiol groups (–SH) was more pronounced in WBM at a wavelength of 2557 cm^−1^, suggesting a more favorable environment for nanocomposite structure formation in this medium. FT-IR analysis also revealed shifts and broadening of the peaks corresponding to different biomolecular groups in each medium. These alterations provide crucial corroborative evidence that supports the discussion on the individual roles of each component in nanocomposite formation. The FT-IR analysis described above aligns with established references in the field, offering substantial insight into the biochemical interactions at play (Fig. 6).

Our investigations revealed that among the strains tested, *Lb delbrueckii* and *Lb acidophilus* exhibited more favorable proliferation in WBM than in MRSB. This observation was made at an initial concentration of 4 mmol AgNO_3_. Furthermore, the size distribution of the metal cores and DLS analysis revealed that AgNCs synthesized from both strains exhibited smaller particle sizes and more uniform dispersion in the WBM than in the MRSB. Considering these findings, it became imperative to investigate the antimicrobial and antioxidant activities of the final product to obtain a comprehensive understanding of its bioavailability. By examining these aspects, we can better understand the potential applications and effectiveness of the final product.

### Antimicrobial activity of the AgNCs

#### The antimicrobial activity of AgNCs

**Fig. 7:**
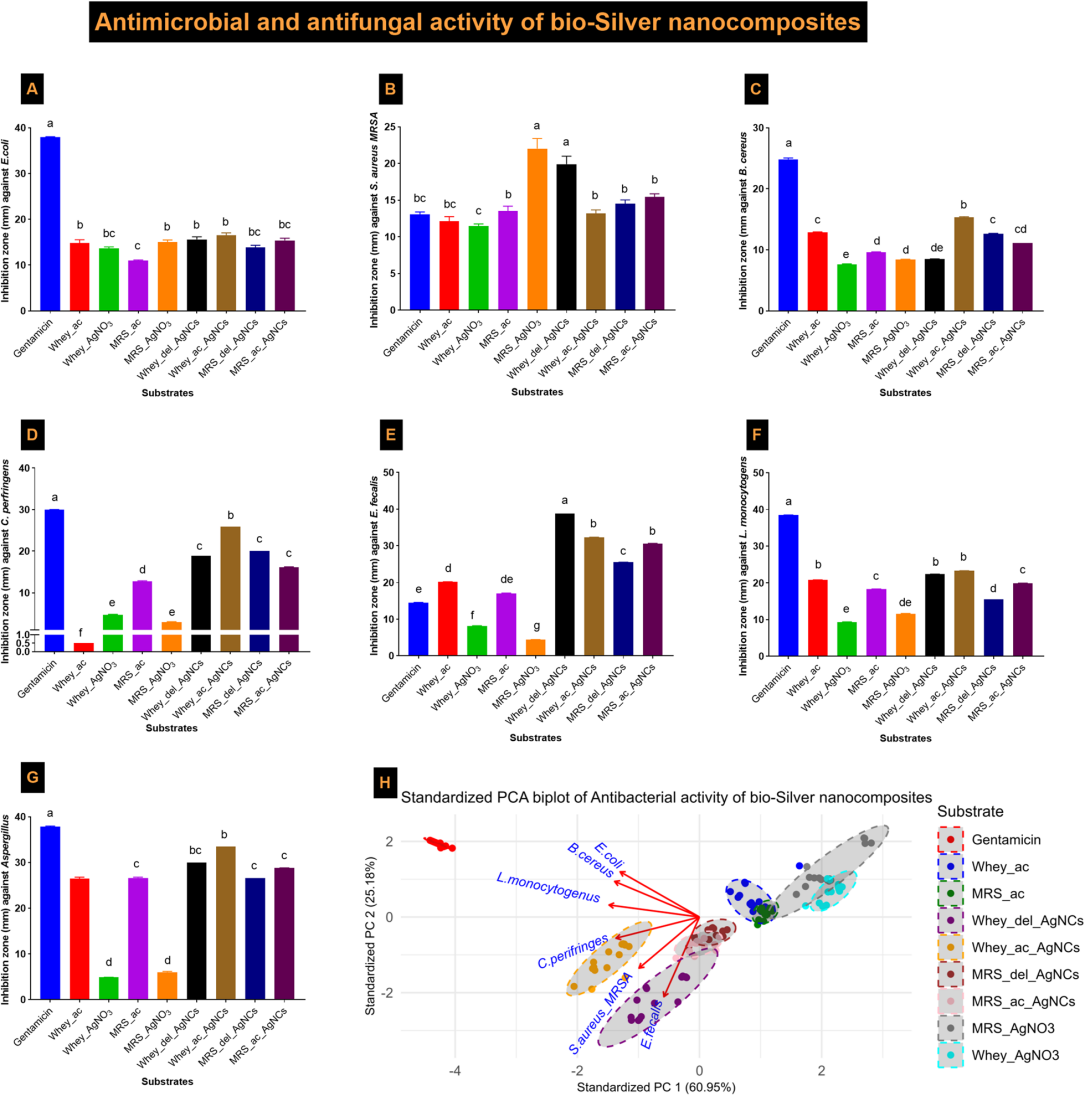
Antibacterial and antifungal activities of AgNCs and PCA of nanocomposite antibacterial modes of action **A** *Escherichia coli*, **B** *Staphylococcus aureus* (MRSA), **C** *Bacillus cereus,* **D** *Clostridium perfringens*, **E** *Enterococcus faecalis*, **F** *Listeria monocytogenes*, and **G** *Aspergillus brasiliensis*. **H** Standardized principal component analysis (PCA) of the similarities in the antimicrobial efficacies of the nanocomposites against bacterial pathogens. Whey-del-AgNCs: Synthesized using *Lb delbrueckii* grown in whey medium with an initial concentration of 4 mmol AgNO_3_. MRS-del-AgNCs: Synthesized using *Lb. delbrueckii* grown in MRS broth medium with an initial concentration of 4 mmol AgNO_3_. Whey-ac-AgNCs: Synthesized using *Lb acidophilus* grown in whey medium with an initial concentration of 4 mmol AgNO_3_. MRS-ac-AgNCs: Synthesized using *L. acidophilus* grown in MRS broth medium with an initial concentration of 4 mmol AgNO_3_

Synthesized using *Lb delbrueckii* and *Lb acidophilus* was evaluated via agar well diffusion, MIC determination, and fluorescence confocal microscopy analysis. The antibacterial activity of AgNCs synthesized from two lactobacilli strains on WBM and MRSB in agar well diffusion assays was investigated against *E coli, B cereus, C perfringens, E faecalis, L monocytogenes,* and methylene-resistant *Staph aureus* (MRSA) and compared to that of the broad-spectrum positive control gentamicin. Both bioactive AgNCs synthesized from WBM exhibited a significantly greater antagonistic effect against all the bacterial pathogenic strains than did the nanocomposites synthesized from MRSB (Fig. 7H A–F). AgNCs efficacy against all the tested pathogenic bacterial strains was 89.73% according to standardized PCA (Fig. 7H). Similarly, both bioactive AgNCs synthesized from WBM had antifungal activity similar to that of terbinafine (0.4 µg/ml), a positive control antifungal agent, followed by nanocomposites synthesized from MRSB standard medium. The results concluded that the AgNCs synthesized using *Lb acidophilus* in WBM had the most aggressive antibacterial and antifungal activity among all the tested samples (Fig. 7G).

#### Minimum inhibitory concentration (MIC)

To better understand the inhibitory effects of AgNCs synthesized in both WBM and MRSB on various pathogenic strains, the MIC values were determined. The investigation revealed that AgNCs exhibited varying degrees of antimicrobial effectiveness against strains such as *E. coli, Staph aureus,* MRSA*, C perfringens, and B cereus,* depending on the synthesis medium. Specifically, the AgNCs had lower MICs in WBM than in MRSB, suggesting that the composition of the medium influences the antimicrobial potency of the AgNCs (Table 2).

**Table 2:** Minimum Inhibitory Concentration (µg/ml) of AgNCs

	C + *	Minimal inhibitory concentrations (µg/ml)			
		W-del-AgNC	M-del-AgNC	W-ac-AgNC	M-ac-AgNC
*E coli* ATCC 8739	8.00	12.5	12.5	6	12.5
*Staph aureus* MRSA ATCC 43300	ND	12.5	25	6	12.5
*Clostridium perfringens* ATCC 13124	5.00	6	12.5	6	3
*Bacillus cereus* ATCC 10876	2.00	3	6.00	3	1.5

^*^C + : Gentamycin, Whey-del-AgNCs: Synthesized using *Lb delbrueckii* grown in whey medium with an initial concentration of 4 mmol AgNO_3_, MRS-del-AgNCs: Synthesized using *Lb delbrueckii* grown in MRS broth medium with an initial concentration of 4 mmol AgNO_3_. Whey-ac-AgNCs: Synthesized using *Lb acidophilus* grown in whey medium with an initial concentration of 4 mmol AgNO_3,_ MRS-ac-AgNCs: Synthesized using *L. acidophilus* grown in MRS broth medium with an initial concentration of 4 mmol AgNO_3_

#### Confocal microscopy analysis

**Fig. 8:**
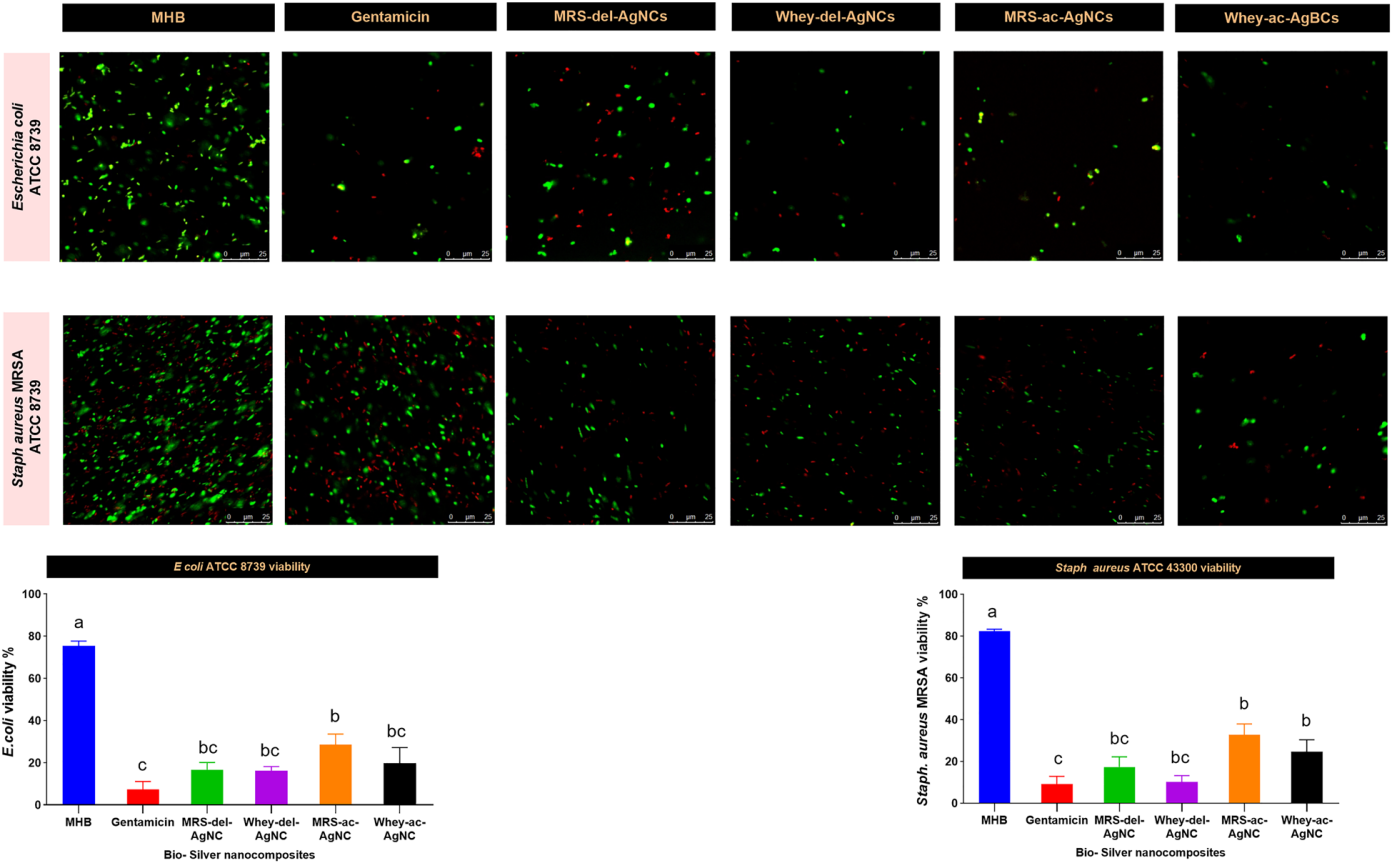
Effects of AgNCs on the viability of *E. coli* and Staph aureus MRSA MHB: untreated pathogenic strains grown in Muller–Hinton broth (negative control) Gentamicin: broad-spectrum antibacterial agent (positive control) Whey-del-AgNCs: Synthesized using *Lb delbrueckii* grown in whey medium with an initial concentration of 4 mmol AgNO_3_ MRS-del-AgNCs: Synthesized using *Lb delbrueckii* grown in MRS broth medium with an initial concentration of 4 mmol AgNO_3_ Whey-ac-AgNCs: Synthesized using *Lb acidophilus* grown in whey medium with an initial concentration of 4 mmol AgNO_3_ MRS-ac-AgNCs: Synthesized using Lb*. acidophilus* strains grown in MRS broth medium with an initial concentration of 4 mmol AgNO_3_

To elucidate the robust antibacterial efficacy of the WBM AgNCs in contrast to those synthesized in MRSB medium utilizing *Lb delbrueckii* and *Lb acidophilus* strains, the bacterial strains *E coli* and *Staph aureus* MRSA were exposed to both AgNCs for 24 h. Subsequently, the AO/EB staining protocol was used to assess the number of viable bacterial cells (%). These findings substantiated our initial observations, underscoring the superior antibacterial performance of WBM AgNCs synthesized from both *Lactobacillus* strains compared to their counterparts in MRSB medium (Fig. 8).

### Antioxidant activity: free radical scavenging assay (RSA)

**Fig. 9:**
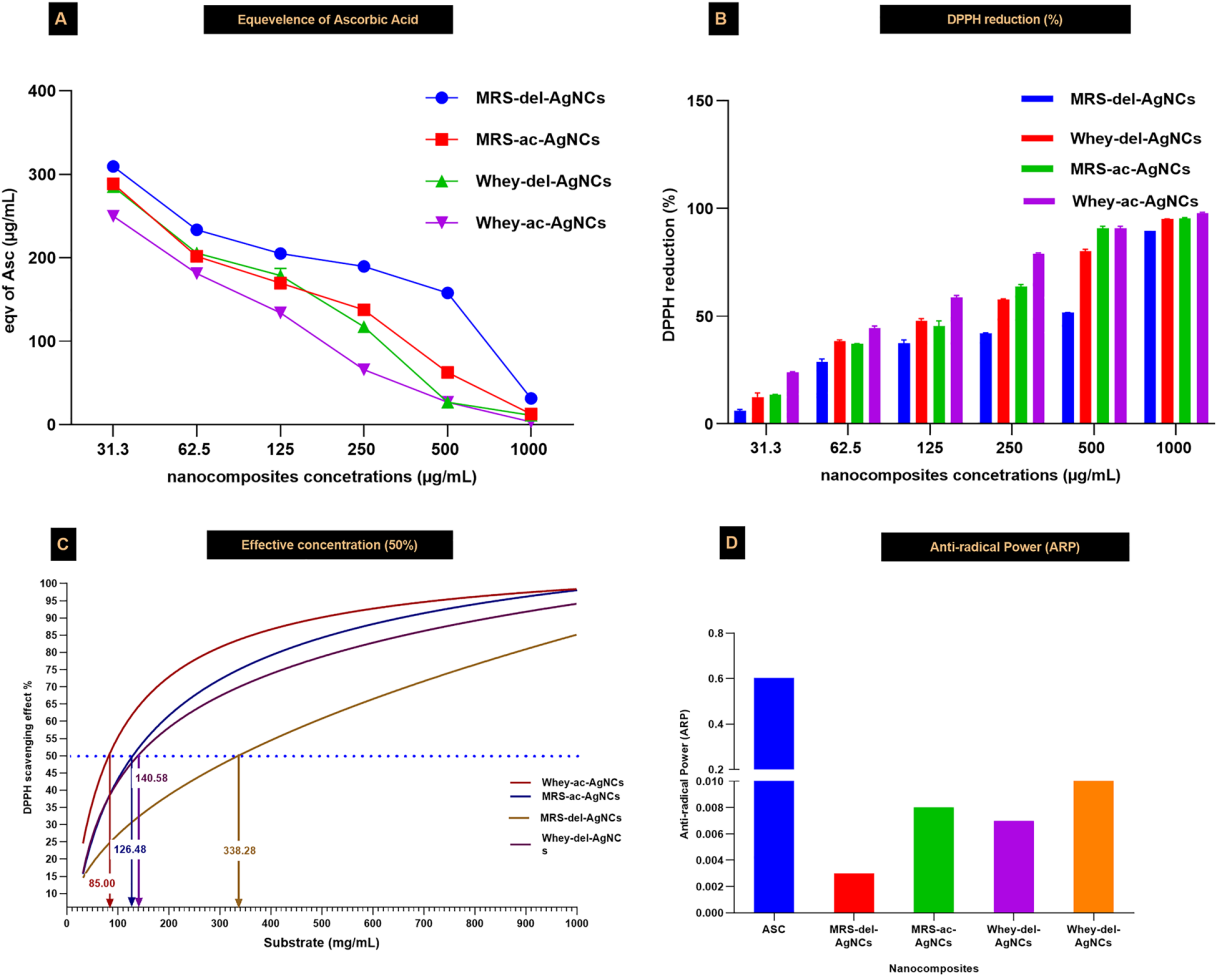
Free radical scavenging of AgNCs in MRSB and WBM compared to ascorbic acid **A** The free radical scavenging activity is expressed as a percentage of the ascorbic acid content of AgNCs and is represented as an equivalent of ascorbic acid according to the DPPH reducing assay. **B** Percentage of DPPH reduction using AgNCs. **C** EC50 concentrations (µg/mL) of **D** the antiradical power of AgNCs from *Lactobacillus* strains in MRSB and WBM compared to that of ascorbic acid. ASC: Ascorbic acid concentration as a positive control Whey-del-AgNCs: Synthesized using *Lb delbrueckii* grown in whey medium with an initial concentration of 4 mmol AgNO_3_ MRS-del-AgNCs: Synthesized using *Lb delbrueckii* grown in MRS broth medium with an initial concentration of 4 mmol AgNO_3_ Whey-ac-AgNCs: Synthesized using *Lb acidophilus* grown in whey medium with an initial concentration of 4 mmol AgNO_3_ MRS-ac-AgNCs: Synthesized using *L. acidophilus* grown in MRS broth medium with an initial concentration of 4 mmol AgNO_3_

The antioxidant capabilities of bioactive AgNCs synthesized from *Lb delbrueckii* and *Lb acidophilus* in WBM and MRSB were evaluated. Ascorbic acid was used as a benchmark antioxidant for comparison. The efficacy of these AgNCs was quantified in terms of their equivalence to ascorbic acid (ASC), with AgNCs derived from *Lb acidophilus* in WBM showing the highest similarity to ASC. This assessment was further substantiated through the determination of the half-maximal effective concentration (EC50) and the percentage reduction of 2,2-diphenyl-1-picrylhydrazyl (DPPH) yield, which indicated that there was a direct correlation between the increase in AgNCs concentration (ranging from 31.3 to 1000 µg/mL) and the increase in DPPH radical scavenging activity. The kinetics of the antioxidant activity revealed that AgNCs produced in MRSB exhibited a slower reaction rate than did their counterparts synthesized in WBM for both bacterial strains. Consequently, the antioxidant reactivity potentials (ARPs) were notably greater for AgNCs from *Lb acidophilus* in the WBM than for those from *Lb delbrueckii* in the WBM; subsequently, AgNCs in the MRSB from both strains were generated when aligned against the reference standard (Fig. 9A–D).

### Ferric reducing power assay (FRAP)

**Fig. 10:**
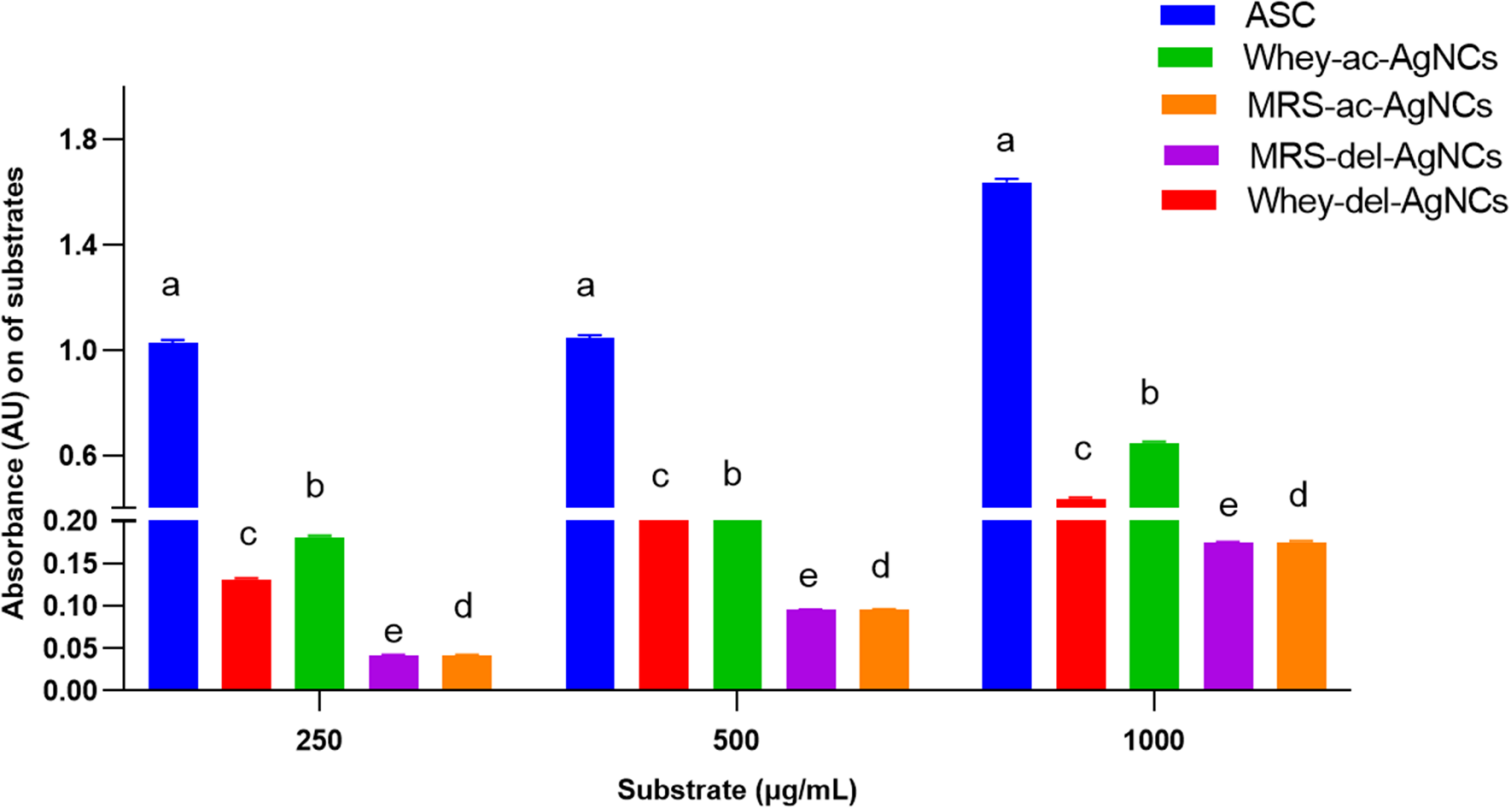
Ferric reducing power of AgNCs in MRSB and WBM compared to ascorbic acid ASC: Ascorbic acid concentration as a positive control Whey-del-AgNCs: Synthesized using *Lb delbrueckii* grown in whey medium with an initial concentration of 4 mmol AgNO_3_ MRS-del-AgNCs: Synthesized using *Lb delbrueckii* grown in MRS broth medium with an initial concentration of 4 mmol AgNO_3_ Whey-ac-AgNCs: Synthesized using *Lb acidophilus* grown in whey medium with an initial concentration of 4 mmol AgNO_3_ MRS-ac-AgNCs: Synthesized using *L. acidophilus* grown in MRS broth medium with an initial concentration of 4 mmol AgNO_3_

To confirm the results, a FRAP assay was conducted. The data confirmed that silver nanocomposites from both tested *lactobacilli* strains on WBM exhibited slower but superior antioxidant activity compared to the same AgNCs from MRSB in the case of both strains (Fig. 10).

## Discussion

This study highlights the potential of synergetic effect of alive lactobacilli metabolites with WBM as an eco-friendly medium on the biosynthesis of AgNCs using five lactobacilli strains (AgNCs). The results suggest that, compared with MRSB, which is commonly used as a standard laboratory medium for growth and biosynthesis, WBM can significantly enhance the growth and biomass yield of all five tested lactobacilli. This is likely due to the unique components in WBM that can optimize the proliferation and productivity of lactobacilli strains during biosynthesis [36–38].

Lactose, the primary carbon source in the WBM, is a crucial factor in the proliferation of lactobacilli cells. In addition, WBM fortification with glucose had enhanced growth and AgNCs biosynthesis in several lactobacilli strains, including *Lb rhamnosus, Lb acidophilus, Lb casei, Lb plantarum, Lb ruminis, Lb paracasei* subsp *paracasei, Lb delbrueckii, Lb helveticus,* and *Lb reuteri* [36, 39]. Another unique component is nutrient-rich peptides (bioactive peptides) and amino acids in whey, which may be present at 0.6–0.8% w/v and stimulate lactobacilli cells abundance [40]. Nitrogen expedites growth and the metabolic rate of lactobacilli, resulting in the production of lactic acid, amino acids, and protein fragments (peptides), which are considered key growth factors for lactobacilli strains [41]. Moreover, stallic acid, a type of oligosaccharide that attaches itself to cellular proteins; lactoferrin, an iron-binding glycoprotein; and calcium in calcium phosphate are enhancing tools that lactobacilli cells can use to maximize their growth rate [42]. These unique components of WBM promote the growth and health of the five tested lactobacilli strains [38]. This hypothesis was confirmed by SEM images, which showed that the cells of the five tested lactobacilli strains had a greater growth rate in the WBM than in the standard MRSB growth medium.

To study the synergetic effect of WBM on the biosynthesis of AgNCs lactobacilli, a comparative study was carried out on the individual growth rates of five strains of lactobacilli after exposure to 0.1 mmol AgNO_3_ via WBM and MRSB. The logarithmic phase of all strains was delayed for 10 h in both media, likely because the strains adapted to the stressor by activating the expression of genes related to silver nitrate tolerance, which might be due to the transcription of genes that produce NADH-dependent enzymes, specifically nitrate reductase enzymes. Lactobacilli detoxify silver nitrate by secreting reducing agents and utilizing their cell membrane functional components to biosynthesize nanocomposites as a potential defense mechanism [43–46]. When the strains were reinoculated at relatively high concentrations, the growth rate of the five strains of induced lactobacilli was much greater than that of the other strains during the induction stage. Moreover, the growth rate of the lactobacilli strains in the WBM was slightly greater than that in the MRSB at all silver nitrate stress levels, confirming the previously discussed hypothesis of the unique nutritional effect of the WBM components [24, 28, 41]. According to our previously mentioned results, the optimum growth rate of the five tested lactobacilli strains after 24 h of incubation at 35 ± 2 °C was approximately 4 mmol AgNO_3_. The electron microscopy results revealed that silver nanoparticles were synthesized on the cytoplasmic cell membrane, within the cytoplasm, and outside the cells. This phenomenon validated the enzymatic reduction of metal ions by enzymes located on the cytoplasmic membrane and within the cytoplasm [24, 47]. In the WBM, the same bacterial cells at the same initial AgNO_3_ concentration might use the lactose–glucose mixture to exert higher concentrations of exopolysaccharides, forming a protective matrix called a biofilm, which might serve a dual function, protecting cells and extracellularly biosynthesizing the bioactive nanocomposite [48]. In other words, the SEM and TEM images clearly showed that lactobacilli cells at the end of the incubation period (72 h) were morphologically damaged in MRSB in response to the initial concentration of AgNO_3_. In contrast, the tested lactobacilli strains utilized WBM components to protect their morphological shapes under the same AgNO_3_ stress, and the damage rates were much lower than those of MRSB during the same incubation period (72 h). A detailed study of the potential defense mechanism(s) utilized by lactobacilli in the WBM is ongoing.

DLS was used to determine the hydrodynamic size distribution of the AgNCs, and the presence of the whey-rich component of the proteins and peptides resulted in larger AgNCs than did the presence of the same products on MRSB. TEM images and particle size distributions, from the second size investigation approach, showed that the nanocomposite silver metal cores were not in contact with each other in the WBM, while agglomeration was more obvious in the MRSB. The size of the bio-nanosilver metal core ranged from 10–77 nm in MRSB but was only 8–46 nm in WBM when the initial concentration of silver nitrate was the same [32, 49–51]. In addition, the TEM image of the AgNCs metal core confirmed that the WBM components functioned as capping agents, facilitating the formation of polydispersed nanometals with spherical and smooth surfaces. The tested strains biosynthesized well-separated, aggregated, and peptide-stabilized silver nanoparticles in the WBM [32, 49]. The DLS was greater than the size measured by TEM, which might be contradictory. However, this could support the idea that organic compounds containing biomolecules serve to cap and stabilize the silver metal core of the nanocomposites [28]. The lactobacilli biomass could be a sufficient reducer for silver nitrate and a capping agent for silver nanoparticles. The resistance of these materials to the toxic effects of silver cations (Ag +) is due to their ability to ligate their membrane functional groups, thereby reducing their resistance and simultaneously composing nanocomposites [52].

In our study, five different *Lactobacillus* strains, *Lb rhamnosus, Lb acidophilus, Lb casei, Lb plantarum,* and *Lb delbrueckii,* were subjected to six concentrations of silver nitrate (1–6 mmol) for 24 h. Each strain achieved the highest biomass yield at varying concentrations based on their biochemical metabolism pathways and unique defense mechanisms [28, 47, 53]. Although the precise biosynthetic mechanism of AgNCs has still not been elucidated, several hypotheses have been proposed to clarify this mechanism. *Lb. plantarum* bacterial biomass might reduce silver cation (Ag^+^) stress via a cytoplasmic enzymatic bio-reduction process [28]. In addition to these reducing enzyme mechanisms, exopolysaccharides secreted by *Lb rhamnosus* may not only be extracellular reductants of Ag^+^ cations to Ag^0^ but also act as capping agents [47, 54]. *Lb acidophilus* and *Lb casei* were found to protect themselves by transporting proteins outside the cell enclosure for the simultaneous biosynthesis of silver nanoparticles and prevention of nanoparticle aggregation [55]. Finally, *the biomass of Lb bulgaricus* has been reported to utilize cell membrane components, *e.g.,* carbonyl groups from amino acid residues and protein peptides, because of their strong ability to bind silver [56]. Moreover, the protein forms a coating to stabilize the metal nanoparticles to ultimately produce highly reduced AgNPs from the oxidized state [57, 58].

The UV/Vis spectral peaks confirmed the organic capping agent(s) hypothesis. In more detail, the surface plasmon resonance (SPR) absorbance of silver nanoparticles ranges from 391–440 nm, whereas the absorbance of proteins and amino acids ranges from 200–290 nm. According to our findings, it was 256–264 nm long, which may be affected by the bioactive AgNCs content of lactobacilli metabolites and the utilized biosynthesis media components, such as proteins, amino acids, and peptides [59, 60]. FTIR validated and characterized the previously indicated functional bio-reducing groups in the biosynthesized lactobacilli-AgNCs. Proteins, aldehydes, esters, alcohols, and amino acids are among the reported functional groups in lactobacilli cell membranes, and secondary metabolites may function as bio-reductants of silver nitrate to AgNPs. Therefore, the peptidic nature of lactobacilli metabolites may explain why the obtained bioactive AgNCs organic coating may consist of amino acids (AA), such as serine, tryptophan, cysteine, leucine, isoleucine, arginine, and asparagine, which exhibited a strong intensity range of 1658–1630 cm^−1^ in the FT-IR WBM biosynthetic media maps [32, 55, 60–62]. The carbonyl groups in functional protein peptides and free amino acids might have strong binding affinities to AgNPs at high levels [63]. This binding occurs through electrostatic interactions between the carbonyl groups and the AgNPs. This interaction helps carbonyl groups coat the AgNPs surface, forming a layer or coating. This coating acts as a bridge among AgNPs, forming larger aggregates, resulting in more stable final bioactive AgNCs in both biosynthetic growth media. Numerous factors, such as the lactobacilli strains utilized in the biosynthesis process, pH levels, and differences between growth media, might influence the slight shift in vibration frequencies [32, 49].

**Fig. 11:**
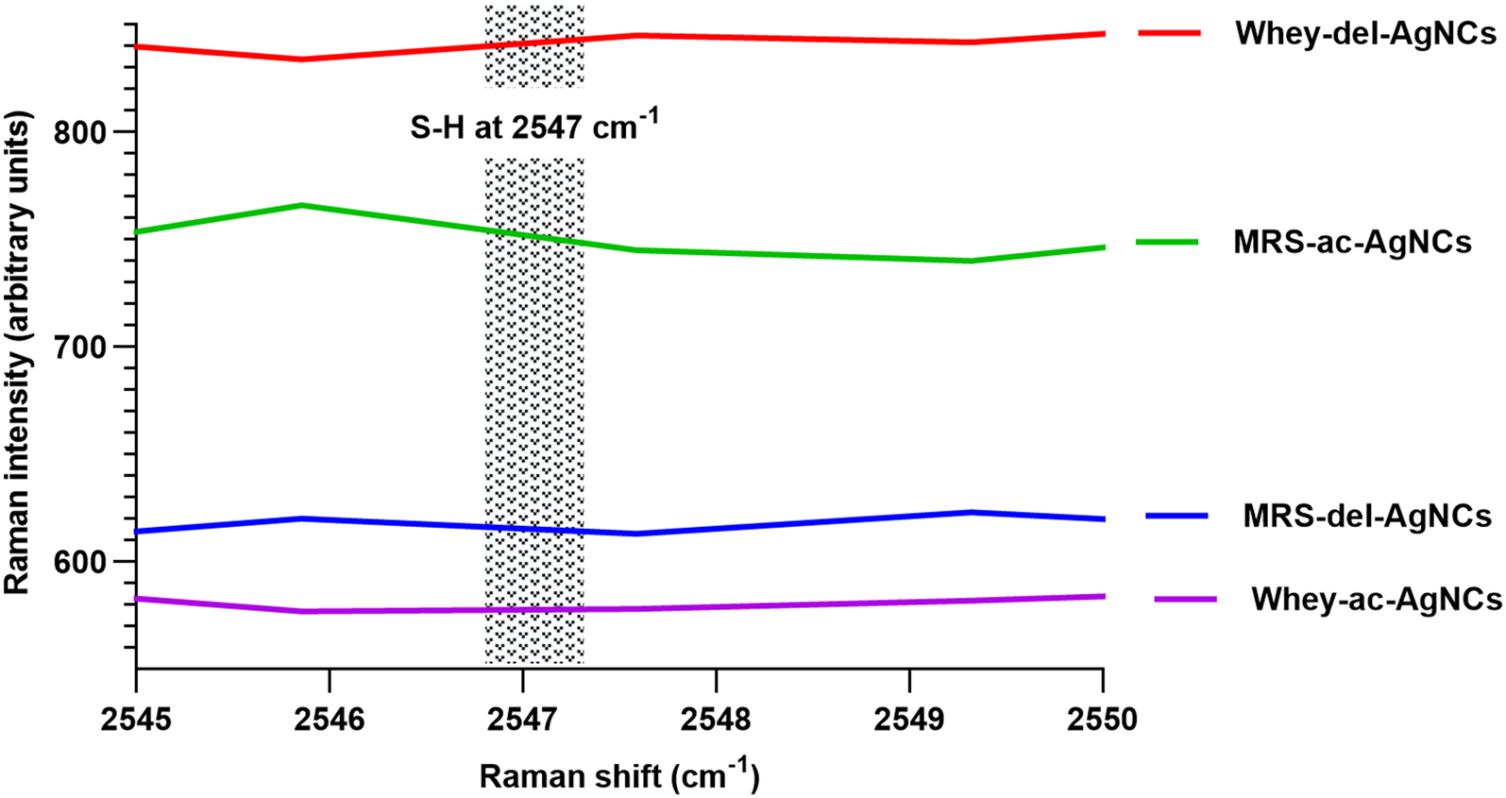
Micro-Raman spectroscopy analysis of AgNCs biosynthesized by *Lb.* *delbrueckii* and *Lb acidophilus* Whey-del-AgNCs: Synthesized using *Lb delbrueckii* grown in whey medium with an initial concentration of 4 mmol AgNO_3_ MRS-del-AgNCs: Synthesized using *Lb delbrueckii* grown in MRS broth medium with an initial concentration of 4 mmol AgNO_3_ Whey-ac-AgNCs: Synthesized using *Lb acidophilus* grown in whey medium with an initial concentration of 4 mmol AgNO_3_ MRS-ac-AgNCs: Synthesized using *L. acidophilus* grown in MRS broth medium with an initial concentration of 4 mmol AgNO_3_

Edx was used to evaluate the elemental composition of AgNCs, and the results revealed the presence of a significant amount of nitrogen, which is the primary component of WBM proteins/peptides. Additionally, sulfur was found in the WBM, specifically the thiol groups in the cysteine amino acid, which is a sulfur-containing functional group. The cysteine active sites are the amine, carboxylic, and sulfur groups. These groups have electron-rich atoms, such as nitrogen, oxygen, and sulfuric acid, which supports the hypothesis that the nanocomposites were synthesized using peptides with disulfide linkages, which are likely protective biocoating agents for the biosynthesized AgNCs metal core [64–67]., Raman spectroscopy confirmed the hypothsis, and the absence of the S–H stretching (2547 cm^−1^) peak indicated S–Ag covalent interactions [30]. These findings are consistent with the FT-IR results, where the S–H stretching band at 2557 cm^−1^ intensity varied among inoculated MRSB, WBM, and free media. Notably, this band disappeared in both media with silver nitrate (Fig. 11).

All the results confirmed the synergetic effect of bacterial growth metabolites and protein/peptide whey components in the formation of an organic coating on silver nanoparticles, known as biocolloids. These biocolloids exhibit a negative charge on their surface. As a result, the protonation and deprotonation states of these biomolecules influence layer diffusion processes, which are crucial aspects that lead to significant structural changes [32, 68]. The surface charge density is correlated with the zeta potential (ζ). It's important to clarify that the zeta potential values alone, being less than ± 30 mV, may suggest limited colloidal stability of AgNCs. However, stability is not solely dictated by zeta potential magnitude. The peptidic nature of the composite coating, mainly cysteine, can impart stability through Ag–S bonding. This chemisorptive interaction provides significant steric and electrostatic stabilization, which is not directly reflected in the zeta potential values. These factors, along with environmental conditions such as pH and ionic strength, collectively influence AgNCs stability. Research indicates that Ag–S bonds enhance nanoparticle stability via strong metal-sulfur interactions, even when zeta potential values suggest marginal stability [69–71] Therefore, a multifaceted approach to assessing stability, beyond zeta potential, is essential for a comprehensive understanding. These factors, in turn, might have implications for the optical properties, hydrophobicity, and reactivity of bioactive AgNCs. Furthermore, understanding the bioactive AgNCs dispersion stability and aggregation and adhesion properties is crucial for comprehending the antiradical and antimicrobial activities of AgNCs. The dispersion behavior of the nanoparticles, their tendency to aggregate, and their ability to adhere to surfaces all contribute to their overall functionality and effectiveness in biological applications. This knowledge can further contribute to developing innovative applications and approaches in various fields, such as nutraceuticals, medicine, materials science, and environmental remediation.

### Potential antimicrobial and antiradical bioactivity

To obtain unambiguous evidence of AgNCs bioactivity, antimicrobial and antioxidant levels were investigated. The inhibitory effects of the bioactive AgNCs synthesized using *Lb delbrueckii* and *Lb acidophilus* in WBM compared to those in MRSB standard medium were tested against *E coli* (Gve-), *B cereus* (aerobic), *Cl perferinges* (anaerobic) spore-formers, MDR *Staph aureus* (MRSA) (Gve +) and *Asp brsillensis* (filamentous fungi) were tested in five individual experiments. The antimicrobial (antibacterial and antifungal) activity of both tested AgNCs produced in WBM was significantly clearer than that of the same AgNCs synthesized using MRSB. Particle size distribution TEM images analysis is one of the parameters that might illustrate what could happen; WBM-enhanced surface modifications or coatings may improve AgNCs stability and prevent aggregation, resulting in nanocomposite metal-core multidispersion, less aggregation, and a smaller particle size. The final larger surface area could contact and attach to the tested pathogenic cell walls, penetrating the protoplasm and acting as a poison that denatures proteins and nucleic acid [72, 73]. This expectation is important, particularly when discussing the AgNCs antibacterial ability to destroy MDR strains such as methicillin-resistant *Staph aureus* (MRSA). MRSA which is a type of *Staph aureus* that is resistant to multiple antibiotics, making it challenging to treat. MRSA often forms biofilms, which are structured communities of bacteria embedded within a protective extracellular matrix. Peptide-silver nanocomposites might disrupt biofilms formed by MRSA [74]. AgNCs can penetrate through the biofilm matrix and target bacterial cells, while silver nanoparticles contribute to the destruction of the biofilm structure and inhibit biofilm formation, suggesting a promising approach for combating MRSA infections. This synergistic effect might also have well-known mechanisms of action involving cell membrane disruption, interference with cellular functions, oxidative stress, and DNA damage, which overcome antibiotic resistance in MRSA strains. Although bioactive AgNCs show promise in combating MRSA strains, further research is needed to understand the optimal conditions, dosage, nanoparticle size, peptide/protein-nanoparticle ratio, and nanocomposite stability [75]. These factors can impact the efficacy and selectivity of the nanocomposite against MRSA and the potential side effects associated with its use.

The AgNCs might exhibit antimicrobial activity against several bacterial spores, including those formed by aerobic *B cereus* and anerobic *C perfringens*. In addition to the aforementioned well-known antimicrobial mode of action of AgNCs may interfere with the germination of bacterial spores, disrupt this process, prevent spores from returning to their active state, attach to the surface of bacterial spores and disrupt the integrity of the spores’ coat and inner membrane. This disruption can affect the resistance properties of spores and weaken their structure Leading affect the early stages of spore development or sporulation. This could lead to fewer spores being produced first. The antifungal mode of action of AgNCs generally involves similar mechanisms, as mentioned earlier. In addition, filamentous fungi can form biofilms, complex structures of fungal cells embedded in a matrix providing increased resistance to antifungal treatments [76]. AgNCs can penetrate the biofilm matrix, inhibit biofilm-associated fungal cell growth, and promote structure dispersal [77, 78]. Moreover, bioactive AgNCs can cause morphological changes in fungal cells, leading to abnormalities in hyphal growth, spore germination, and reproductive structure formation; these changes might ultimately hinder the spread and proliferation of the fungus [79]. In a nutshell, the antagonistic effect can vary depending on the specific microbial species, and the unique characteristics, concentration, size of AgNCs, exposure time, and environmental conditions can also influence the effectiveness of the antimicrobial activity. Further research is ongoing to improve the understanding of the specific molecular interactions and potential synergistic effects of bioactive AgNCs as antimicrobial agents, develop safe and effective strategies for treatments, and mitigate microbial infections.

### Antioxidant activity

It was important to test the antioxidant capacity of the nanocomposites to confirm their bioavailability, as free radicals can cause damage to cells and tissues. Although WBM has a weak antioxidant effect, it contains certain compounds with powerful antioxidant effects [80]. Cysteine is a precursor to glutathione, which is a crucial antioxidant component [81]. Bioactive peptides, including lactoferrin immunoglobulins and lactokinins. [82, 83]. Vitamins, minerals, and enzymes such as vitamin C and vitamin E) and minerals (e.g., selenium and zinc) that act as cofactors for antioxidant enzymes such as lactoperoxidase are crucial components of WBM that could aid in its overall antioxidant capacity [84]. Our DPPH and FRAP results revealed that lactobacilli strains enhanced the antioxidant activity of WBM, as they might produce peptides and bioactive components, including γ-glutamyl cysteine [85–87]. Moreover, nanocomposites comprising lactobacilli fermentation products and silver nanoparticles could increase the free radical scavenging capacity of the final bioactive AgNCs [32]. Bioactive AgNCs, due to their high surface area, possess intrinsic antioxidant properties, where they can interact with and neutralize reactive oxygen species (ROS), reducing oxidative stress and preventing harmful effects. Furthermore, they can increase the activity of endogenous antioxidants such as superoxide dismutase (SOD) and catalase (CAT), leading to increased expression and activity of antioxidant enzymes. Additionally, bioactive AgNCs can effectively safeguard DNA against oxidative damage [88–90]. The effectiveness of antioxidant mechanisms is influenced by various factors, such as the chosen method and assay, reaction time, and medium. The properties of silver and bacterial strains can also play a role in interactions with free radicals. Therefore, metal silver nanoparticles and organic surfaces containing multiple amino acids may contribute to this capacity. Therefore, bioactive AgNCs can act to neutralize and scavenge ROS, protecting cells and tissues from oxidative damage [91, 92]. Notably, the apparent contradiction between the antimicrobial and antioxidant modes of action of silver nanocomposites can be reconciled by understanding the nuanced mechanisms involved and the context in which these nanocomposites operate. Several factors that might explain how AgNCs can exhibit both properties include selective toxicity [93, 94], controlled release and activation [95], surface modification and coating [96], dose dependency [97], and environmental conditions [98].

## Conclusion

This research demonstrates the synergistic effects of *Lactobacillus* strains in a whey-based medium for the efficient biosynthesis of silver nanocomposites (AgNCs). Leveraging the inherent properties of lactobacilli, the study enhances both the antimicrobial and antioxidant capabilities of AgNCs. Advanced characterization techniques revealed significant antimicrobial activity against a broad spectrum of pathogens, including multidrug-resistant strains, addressing key concerns in antimicrobial resistance.

The use of whey, a sustainable byproduct, underscores the environmental benefits and enhances the stability of the nanoparticles. These findings suggest significant utility for AgNCs in medical and nutritional applications, offering a promising approach to developing new antimicrobial agents that are both effective and environmentally friendly. This study paves the way for further exploration of bio-based methods for nanoparticle synthesis with potential broader applications in healthcare and industry.

## Data Availability

This manuscript does not report data generation or analysis.
